# Traditional ecological knowledge sustains due to poverty and lack of choices rather than thinking about the environment

**DOI:** 10.1186/s13002-023-00640-1

**Published:** 2023-12-19

**Authors:** Abdullah Abdullah, Shujaul Mulk Khan

**Affiliations:** 1 Department of Plant Sciences, Quaid-i-Azam University Islamabad, Islamabad, Pakistan; 2 Pakistan Academy of Sciences, Islamabad, Pakistan; 3 University of the Gastronomic Sciences Pollenzo, Pollentia, Italy

**Keywords:** Conservation, Extinction, Lack of resources, Marginalized tribal communities, Medicinal and multiprupose utilizations, Sustainability, Traditional ecological knowledge

## Abstract

In this debate article, we have tried to discuss Traditional Ecological Knowledge (TEK) and its close link to the available resources and lack of options in the indigenous communities. We came across the article of Hartel et al. where they initiated a discussion on this important but debatable topic of TEK and its merits and demirits for the environment. We believe that this discourse could continue to clarify both sides of the picture. Our research group is working on species facing extinction threats due to extensive utilization, leading to overexploitation of the taxa, which the TEK seldom cares for. We are of the opinion that the marginalized tribal communities living around the globe extensively use some of the species and natural resources for their food, fodder, fiber, shelter, energy, health and other benefits, irrespective of their conservation needs, and many other ecosystem services. They have to sustain their basic live’s needs from the resources available for their livelihood. They collect economically important medicinal and other species in large quantities to gain higher earnings, rather than thinking of the issues faced by them. Hence, we believe that the continuity and utilization of TEK are driven by poverty and lack of choices rather than positive environmental values, historically. Nevertheless, those communities sometimes have their local system of protection which sometimes works very well or otherwise with the passage of time and the formation of government-driven regulations in the recent past, TEK itself has lost its real sense especially, related to conservation and management. Therefore, TEK could be linked with existing research-based policies and regulations for long-lasting benefits to the environment and its sustainable future. Such bridging can benifit all the stake hoders—the TEK holders, regulatory bodies, government agencies and many more. This debate can lead to a positive and critical discourse towards the clarification of the conundrum under consideration as well as creation of more questions hyptheses related to TEK.

## Introduction

We are writing this debate in favor of the postulate arguing, "sometimes TEK (Traditional Ecological Knowledge) is maintained not because of positive values about the environment, but because of poverty and a lack of options" written by Hartel et al. [1]. Historically, humans have been hunters and collectors of different animal and plant species for their needs [2], often without considering their environmental values. In rural communities, human beings have a strong bond with nature and natural resources; owing to their availability worldwide. For instance, the traditional societies of the Hindu-Himalayas (but not limited to) have been engaged in hunting, poaching and the consumption of different species since time immemorial. Simultaneously, botanists and herbalists have been involved for centuries in botanical science, especially considering TEK, with a longstanding history. However, the shortage or higher demand for a natural resource, for instance a species; consistently compelled traditional societies to think about their protection and conservation which sometimes works as awareness for environmental values. Yet, practical implementation of such awareness has sometimes been hindered by societal conflicts and competition for the very kind of resource or species [3].

Many ethno-biologists believe that TEK plays a crucial role in safeguarding resources. Yes, there is no doubt about it, but with the slowly and gradually diminishing TEK, some segments were lost completely, some partially, and others existed. As custodians of TEK, we think that TEK related to resource utilization is sustained very well, but a part related to conservation is diminished. Disappearing of the conservation part of TEK could be linked to the exponentioal growth of human population that led to limitations of resources, increease in demands and ultimately competition among the TEK holders. 

Moreover, due to the increasing human population worldwide [4], there is a need for enormous resources. Most requirements in rural societies are fulfilled by the surrounding ecosystem using TEK [5]. Mountains, like many other ecosystems, are considered hotspots for biodiversity as well as TEK [6]. The younger people following their parents' practices on how to use different biological resources ensures the cultural transmission and sustainability of TEK from generation to generation [7, 8]. People use plants and other natural resources for therapeutics, food, energy, nutrition, fuel, construction, clothing, fibers, ropes, utensils, esthetics and many other purposes.[9]. There has always been pressure from urban societies, especially richer people, on these resources.

On the other hand, TEK has long been enjoyed for its tremendous potential in the promotion of sustainable environmental practices and cultural values [10–12]. TEK is sometimes considered an important tool for the conservation and management of ecosystems and biological resources [13]. We support this statement to a limited extent where TEK revolves around basic benefits rather than overall environmental positives, especially in the developing world and modern trends of globalization. We quote an example from Pashtun society (including about 60 million people), where they try to maintain their natural resources if experience a shortage or risk to it. For instance, forest trees, especially various species of gymnosperm, are protected for future generations via social rules known as the Jirga system. Individuals are forced to pay a fine locally referred to as “Nagha” or “Manda” to the community of the share holders of the forest if they violate the rules by cutting down a tree or exceeding the permissible limits where there is a conservation risk. The community gathers in a traditional manner known as ‘Jirga’ (a sort of assembly). Decisions taken by the Jirga are respected and obeyed by all people [14]. It has a positive value for concrete benefits or provisioning ecosystem services, but it is closely related to the shortage of resources rather than other types of environmental services, like regulating and esthetic services. On the other hand, developed nations think about other environmental values including cultural, regulating and supporting services as well. Being part of a developing society, we are convinced that a society that has access to basic needs can think about environmental values but faces deficiencies in TEK and vice versa.

That is why, we believe that TEK is not always maintained as a conscious choice driven by positive environmental values but as one of the survival strategy in the face of socioeconomic challenges [15]. Poverty and lack of options significantly influence individuals as well as societies to get involved in ecologically unsustainable practices. In many regions of the world, particularly in Asia, Africa, and South America remote and marginalized societies often face problems due to a lack of resources, health facilities, access to education and infrastructure, despite having rich TEK. TEK offers them a means of survival under such circumstances, with limited resources. Economic pressure is another factor that leads traditional communities to rely on TEK for their livelihood without considering the environmental values. This pressure is turned into an opportunity for them where the richer offer considerably handsome packages for buying TEK-based products. To support our argument, we are going to describe three examples that is case (I) a bird species, case (II) plant species and case (III) abandonment of Alp valleys.

## Case I

The Asian Houbara Bustard (*Chlamydotis undulata* Jacquin) bird faces extinction due to its higher demand for meat which is considered an aphrodisiac by TEK holders. The species has been declared vulnerable by the IUCN [16], with a declining rate of 30–49% over three generations [17]. A subset of the total population of the bird migrates among various countries, including the desert parts of Pakistan, with an estimated population of 2500 individuals during winter [18]. Its population has been critically endangered on the Arabian Peninsula because of the property of its meat. Therefore, royal Arabs have been visiting the Pakistani deserts for the last few decades to hunt this bird. They got legal permission and hunt it; for instance, in 2014, the Saudi Prince and his friends hunted approximately 2100 birds. Apart from permitted hunting, the traditional poor poachers and falconers also receive high prices from rich Arabs, which ultimately causes a serious threat to the Bird population. According to an estimation, 3000–14000 Houbara Bustard individuals were illegally caught and transported to Gulf countries [19]. The locals also hunt to meet their dietary needs and also sell them to the rich of the region.

The exceeding demand for this bird species among the rich Arabs and the elite of the society is also one of the major causes of its depletion or vulnerability in the environment.

We tend to argue in favor of the statement that TEK is maintained not because of positive values about the environment but because of poverty and a lack of options through this case. Dwellers of deserts have fewer livelihood opportunities and hence adapt legal as well as illegal hunting and poaching procedures  as source of their earnings, food, and cause decline in the Houbara Bustard’s population.

## Case II

Mazri Palm* (Nannorrhops ritchieana* Griffith, Aitch*)* is an important palm species with a restricted distribution in the Saharo-Arabian and parts of the Irano-Turanian regions [20, 21]. The species is used by poor masses of society to manufacture traditional handicrafts and utilizations for other purposes. More than 30 different handicrafts are processed from its leaves, which play an important role in their livelihoods [22]. Its handicrafts are widely available in different markets and valued for their cultural and esthetic uses. Hence, this species is reported as an important component of provisioning as well as cultural services. Its higher external demand subject it to higher harvesting pressure and overexploitation. The Government of Pakistan passed a legislative Act for its conservation, called the Kohat Mazri Control Act in 1954, with the main objective of protecting, preserving and propagating this Palm species in the region. Conversion of the Mazri Palm Forest into agricultural fields was banned through laws and rules in the region under this Act [23]. It helped a lot in the region where the act was implemented with true spirit. On the other hand this law had not been implemented in all parts of the tribal belt, which led to a continuous and overharvested utilization of the species for handicraft formation in the remaining regions. The region has been highly affected by tribal wars and wars for decades in adjacent Afghanistan and hence most of the people live under the poverty line. They use the species continuously to handle their poverty without thinking about its environmental values, though the species is on the verge of endangerment. Globalization, industrialization, and the increase in choices have recently been of more help in the form of available and cheaper alternatives to Mazri-made handicrafts, in protection of the species but eroding the TEK slowly and gradually, at the same time too. Here, it is quite clear that it is government law rather than TEK that protects the exploitative Mazri collection.

## Case III

In many Alps valleys such as of Germany, France, Italy, Switzerland, Hungary and Austria (North Pole), people have abandoned the mountainous lands that were traditionally used for grazing medicinal plants, fuels and fodder collection, based on their long-lasting TEK. These abandonments have a positive impact on the environment in general and on plants and animal species in particular. On the other hand, in the Hindu Kush & Himalayas’ valleys (Southern Pole), these abandonment practices seldom occurred and hence the animals graze on ecologically important plant species and human continue to harvest that pose serious threats to the the species and other natural resources. This strengthens our claim that TEK is maintained only because of the lack of choices. Here, it is also evident that the abandonment of the Alps valleys indicates the recent facilitation of the people, decline in TEK, & enforcement of land use policies; and hence the resultant increase in plant and animal populations and a pollution-free ecosystem that supports our argument [24–26].

In addition to the specific cases discussed above, many other plant species are facing extinction problems owing to their unique medicinal and multi-purpose potentials, leading them to overexploitation. Some of these are included in the Red List by the International Union for Conservation of Nature (IUCN). For instance, *Gentiana kurroo* Royle, *Saussurea costus* (Falc.) Lipsch, *Aconitum chasmanthum* Stapf), and *Lilium polyphyllum* D. tubers are collected for use in the herbal industry for their medicinal and economic values leading them to extinction and are classified as critically endangered by IUCN [27, 28]. *Saussurea costus* is uprooted for its high economic demand and has been in the economic herbal export of the Kashmir region since the colonial period [28]. Several other species for instance *Withania somnifera* (L.) Dunal, *Skimmia laureola* (DC.) Decne, *Juglans regia* L, *Myrtus communis* L, *Zanthoxylum armatum* DC, *Berberis lycium* Royle, *Parrotiopsis jacquemontiana* Rehder, etc., face the problem of overexploitation for medicinal, food, economic and cultural values. The loss of such important species is absolutely against environmental laws such as Khyber Pakhtunkhwa, Forest Ordinance, The Endangered Species Act, and the Convention on International Trade in Endangered Species of Wild Fauna and Flora (CITES) but is the only choice for local communities. People of those remote regions lack alternatives for their health, food and livelihood and hence utilize such species for their needs. External demands and high prices in the market and herbal industries are other important threats to the population of these species [29]. A substantial quantity of these species is transported by TEK holders from rural to urban areas which in turn builds pressure on those species. Therefore, we are convinced that this debate could help in understanding the use of TEK as a lack of options and prevailing poverty in addition to greedy power of purchase by the richest.

We also support the postulate with an interesting example; during the COVID pandemic, herbalists recommended different plant species including *Senna alexandrina* Mill, locally known as Sanna Maki, which was considered the most potential remedy [30]. The price of 100 grams of *Senna alexandrina* before the pandemic was 200 Pakistani Rupees (PKR), with a higher market demand; its price jumped from 200 to 1000 PKR [31]. This species was collected in vast amounts because of its higher demand in the market without thinking of its environmental values. There was no choice of alternatives (vaccines and antiviral drugs) in 2020. However, very few antipyretic/analgesic plants are available as better allopathic drugs[32].

TEK offers remarkable adaptability in changing environmental circumstances, especially with limited resources, poverty, a lack of options as well and access to wealth. This adaptability and resilience stems from needs, greed and desires, rather than thinking about the environment. Indigenous societies use their TEK as well as available resources to make their lives more comfortable and easier. Unfortunately, they compete quite often and over-exploit biological resources to fulfill their economic needs, especially from plants and animals, which ultimately leads to their vulnerability or extinction. In the current economic paradigm, a wealthier society is the main cause of excessive demand, which leads to overexploitation and jeopardizing threatened species that need to be conserved. Moreover, wealthier people pay a substantial amount for collection to traditional societies that grapple with economic challenges. Due to poverty and lack of alternatives, the local poors generate money through their centuries-old TEK and seldom think for the long term impacts of overexploitation of the resources.

### The TEK evaporates when the poor get richer

The TEK holders and the richer segment of the society could jointly be considered responsible for the ruthless utilization of the different species and other natural resources and consequenent causees of a negative impact on the environment.

People while becoming richer, more urbanized and facilitated, push the TEK on the verge of erosion. The economic development of traditional societies causes a disconnection from nature. One of the major causes of the loss of traditional knowledge and practices is a weakening bond with the environment. The wealthy people of society mostly lives in the urban cities and pays a substantial amount to the poors of rural areas to buy products of natural resources inspite of having weaker bonds with nature and natural resources. Prioritization for modern education, development of allopathic medicine, introduction of powerful media and advertisement of synthetic alternatives have wiped out the roots of traditional knowledge over the last several decades. In the indigenous societies, people use various plant species, i.e., *Paeonia emodi* Royle for backache, Rheum australe D.Don, *Withania somnifera* (L.) Dunal as tonic and *Bergenia ciliata* (Haw.) Sternb. for the removal of kidney stones in their traditional ways, where people lack access to modern medicine and other facilities. In the current era, in modern educational institutions, both teachers and students have insufficient information about TEK. Therefore, a continuously widening gap between educated people and TEK holders exists and cause unorganized utilization of the ecosystem services. The situation is further worsened by urbanization, an increase in human population, and migrations; especially, in the developing countries. Additionally, the natural hazards and climatic changes have further weakened the bond between TEK, traditional and modern societies. These factors collectively cause the evaporation of TEK in wealthier communities and exacerbate the pressure on the poor for overexploitation in return [29, 33, 34].

The hypotheses given in the article [1] are under consideration in this debate, especially hypothesis 3 ‘corrupt intent’ and 4 ‘cultural dilution’; We can neither stop the corrupt system completely nor dilute culture with migrations. Therefore, we are supposed to solve the conundrum by adapting the hypothesis, where the author hypothesized externally imposed rules and paradigms. Owing to globalization, overwhelming communications, industrialization, and lifestyle changes, we are convinced that TEK cannot be sustained in its current form. Therefore, TEK could be merged with practicing laws and rules, whether national or international, for better and more sustainable management of both natural resources and the environment. Such transitions are also imperative for TEK itself and its sustainable transformation to the coming generations. The TEK could be guided in the era of highly influencing internet-based media to an entity that is acceptable to the wider range of people in general and policymakers in particular. We are also of the opinion that the debate must be continued that can possibly lead to an hormoney between TEK, modern day science, policies for better solutions and creation of knowledge base prosperous societies.

## Data Availability

Not applicable.

## Abbreviations

TEK: Traditional ecological knowledge
PKR: Pakistani rupees
CITES: Convention on International Trade in Endangered Species of Wild Fauna and Flora
IUCN: International Union for Conservation of Nature
